# Pressure‐Induced Superconductivity in HgTe Single‐Crystal Film

**DOI:** 10.1002/advs.202200590

**Published:** 2022-04-25

**Authors:** Qiang Li, Jian Zhang, Qunfei Zheng, Wenyu Guo, Jiangming Cao, Meiling Jin, Xingyu Zhang, Nana Li, Yanhui Wu, Xiang Ye, Pingping Chen, Jinlong Zhu, Tao Wang, Wangzhou Shi, Feifei Wang, Wenge Yang, Xiaomei Qin

**Affiliations:** ^1^ Department of Physics Shanghai Normal University Shanghai 200234 China; ^2^ Center for High Pressure Science and Technology Advanced Research (HPSTAR) Shanghai 201203 China; ^3^ State Key Lab of Infrared Physics Shanghai Institute of Technical Physics Chinese Academy of Sciences Shanghai 200083 China; ^4^ Department of Physics Southern University of Science and Technology Shenzhen 518055 China

**Keywords:** high pressure, superconductivity, topological property, transporting

## Abstract

HgTe film is widely used for quantum Hall well studies and devices, as it has unique properties, like band gap inversion, carrier‐type switch, and topological evolution depending on the film thickness modulation near the so‐called critical thickness (63.5 Å), while its counterpart bulk materials do not hold these nontrivial properties at ambient pressure. Here, much richer transport properties emerging in bulk HgTe crystal through pressure‐tuning are reported. Not only the above‐mentioned abnormal properties can be realized in a 400 nm thick bulk HgTe single crystal, but superconductivity is also discovered in a series of high‐pressure phases. Combining crystal structure, electrical transport, and Hall coefficient measurements, a p‐n carrier type switching is observed in the first high‐pressure cinnabar phase. Superconductivity emerges after the semiconductor‐to‐metal transition at 3.9 GPa and persists up to 54 GPa, crossing four high‐pressure phases with an increased upper critical field. Density functional theory calculations confirm that a surface‐dominated topologic band structure contributes these exotic properties under high pressure. This discovery presents broad and efficient tuning effects by pressure on the lattice structure and electronic modulations compared to the thickness‐dependent critical properties in 2D and 3D topologic insulators and semimetals.

## Graphical abstract

HgTe is widely used for quantum Hall well studies in thin film form. Under pressure, it undergoes a semiconductor‐to‐metal transition (≈3.5 GPa), followed by superconducting behavior up to 54 GPa, across four high pressure phases. Density functional theory calculation confirms that a surface‐dominated topologic band structure contributes the exotic properties under high pressure.

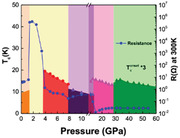

## 1 Introduction

Newly discovered topological insulators (TIs) and topological semimetals (TSMs) have attracted much attention over the last decade due to their unique electronic structures and topological protected surface properties.^[^ ^1^, ^2^, ^3^, ^4^, ^5^ ^]^ The first predicted and discovered 2D TI, HgTe, was fabricated in quantum well form, and its nonlocal transport measurements demonstrate the existence of edge states like the quantum spin Hall insulator.^[^ ^6^ ^]^ When the thickness of the quantum well exceeds the critical thickness of 63.5 Å, the electronic state of HgTe changes from a normal state to an “inverted” type (topological state)^[^ ^7^ ^]^ due to the Γ_6_ and Γ_8_ band ordering reversion. For the 2D HgTe thin film on the CdTe substrate, the topological configuration largely depends on the HgTe‐thickness.^[^ ^8^ ^]^ When the thickness of HgTe layer is below 200 nm, the CdTe substrate can provide enough strain from the lattice mismatch to open the inversion band, as predicted theoretically and observed experimentally.^[^ ^9^ ^]^ For instance, a 65 nm thick HgTe epitaxy grown on a CdTe substrate shows a bulk band gap of 22 meV;^[^ ^10^ ^]^ the quantum Hall effect was observed from the topological surface states of a strained HgTe film with 70 nm thickness.^[^ ^11^ ^]^

With this unique band inversion configuration, bulk HgTe is expected to have a Dirac‐like surface state. As the bulk HgTe material is a semimetal, the surface conduction state is not easy to distinguish from the bulk metal state. Nevertheless, much progress has been made by investigating the surface topological state of both TIs and TSMs. For instance, 3D TI materials Bi_1−_ *_x_* Sb*_x_*, Bi_2_Se_3_, Bi_2_Te_3_, and Sb_2_Te_3_ were proposed and confirmed by angle‐resolved photoemission spectroscopy and scanning tunneling microscopy.^[^ ^12^, ^13^, ^14^, ^15^ ^]^ In high‐quality TIs with a large bulk bandgap and low bulk carrier concentration, the conducting surface state and bulk charge transport can be well separated and identified at low temperature, such as in Bi_2_Te_2_Se^[^ ^16^, ^17^ ^]^ and Bi_1.1_Sb_0.9_Te_2_S.^[^ ^18^, ^19^ ^]^ Increasingly recent investigations have focused on the possible topological superconductivity in 3D TIs and TSMs. Although it remains problematic to clarify the nature of the Cooper pairs existing in the new quantum state in the parent compound, the chemical doping‐induced superconductivity in Cu‐doped Bi_2_Se_3_ has inspired enormous experimental and theoretical efforts to study potential topological superconductivity.^[^ ^20^, ^21^, ^22^ ^]^ An alternative to chemical doping is external pressure, as it can provide a direct way to tune the crystal structure and electronic configuration without introducing additional interferences, such as impurities or vacancies. So far, superconductivities have been reported in several TIs (Bi_2_Te_3_,^[^ ^23^ ^]^ Bi_2_Se_3_^[^ ^24^ ^]^) and TSMs (ZrTe_5_,^[^ ^25^ ^]^ HfTe_5_^[^ ^26^ ^]^) under high pressure. Thus, the transport properties of the HgTe system with its unique topologic band state reversion need to be further explored.

Here, we focus on the evolution of the intrinsic properties of bulk HgTe in high‐quality single‐crystal form with external pressure. A 400 nm thick, single‐crystal HgTe layer was grown on a GaAs substrate with about a 1 µm thick CdTe buffer layer by molecular beam epitaxy (MBE). With in situ high‐pressure Raman and transport measurements, we identified four sequential high‐pressure phases up to 54 GPa, and superconducting behavior in all these high‐pressure phases. Furthermore, the in situ high‐pressure Hall effect reveals a carrier‐type switching from n‐type to p‐type at around 10 K before transforming into a superconducting state in the first high‐pressure phase (cinnabar phase). This work provides an effective pathway to understand the electronic property evolution from thickness‐dependent behavior to pressure‐tuning novel superconductivity in 2D topologic insulators and topologic semimetals.

## 2 Results

### 2.1 Characterization of Single‐Crystal HgTe Layer

**Figure 1:**
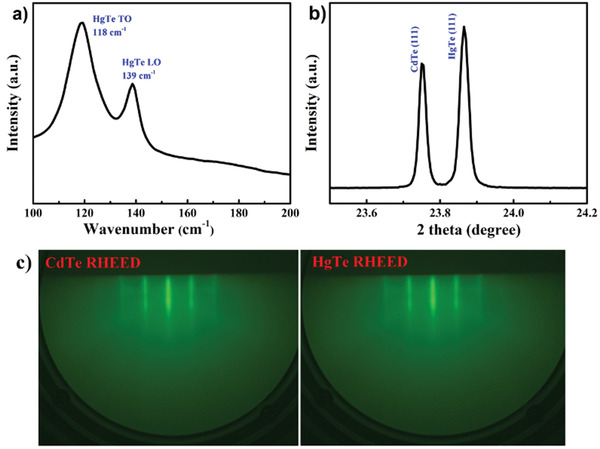
Pristine sample characterization at ambient conditions. a) Raman spectrum, b) X‐ray diffraction, and c) RHEED patterns.

The sharp stripes of the real‐time reflection high‐energy electron diffraction (RHEED) indicate the high quality of the single crystal. The HgTe film (111) may be with smooth surface. X‐ray diffraction and Raman spectra also demonstrate excellent crystal quality, as shown in **Figure** **1**.

### 2.2 High‐Pressure Crystal Structures and Raman Measurements

**Figure 2:**
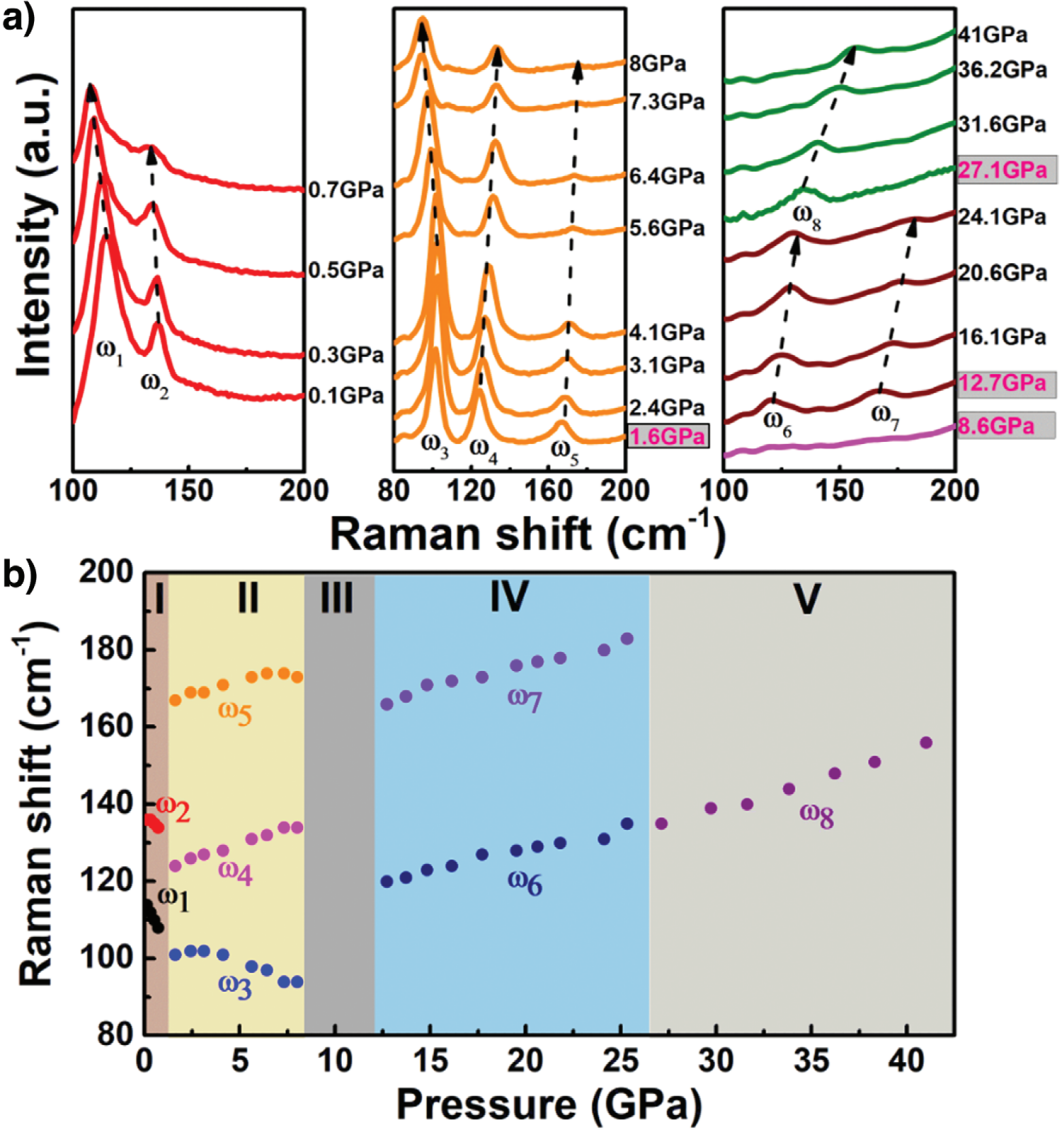
HgTe high‐pressure phase diagram probed by Raman spectroscopy. a) The Raman spectra of HgTe single crystal from 0 to 41 GPa. HgTe undergoes four high‐pressure phase transitions during the compression process, *ω*_1_ − *ω*_8_ are the Raman peak centers during compression. The phase transitions occur at 1.6, 8.6, 12.7, and 27.1 GPa, highlighted in pink color. b) Raman peak positions as a function of the pressure of the HgTe single crystal, which clearly shows five phases in the pressure range of 0–41 GPa.

HgTe crystalizes into a zinc‐blende (phase I) structure at ambient conditions. Under pressure, it undergoes a rich phase transition.^[^ ^27^, ^28^, ^29^ ^]^ The in situ high‐pressure Raman spectra collected from HgTe film in a diamond anvil cell (DAC) at room temperature indicate four high‐pressure phases up to 41 GPa, which matches the previous structure study with high‐pressure X‐ray diffraction (XRD) well. As shown in **Figure** **2**a, at ambient conditions HgTe has two Raman modes at 118 and 138 cm^−1^ associated with the transverse‐optical (TO) and longitudinal‐optical phonons, respectively.^[^ ^30^, ^31^ ^]^ When the applied pressure reached 1.6 GPa, three Raman vibration modes appeared, which matches the HgTe cinnabar phase (phase II) as described by Miller et al.^[^ ^32^ ^]^ All Raman peaks disappeared in the pressure range of 8.6–12 GPa, demonstrating the phase transformation of HgTe from the cinnabar phase to a NaCl‐type nonpolar metallic phase (phase III).^[^ ^27^, ^33^ ^]^ The total silence of Raman modes in phase III further indicates the high‐quality crystallinity of our thin film, as defects or vacancies could enable the Raman modes. Upon further compression, two Raman peaks started to appear at 12.7 GPa, and only one remained after 27.1 GPa, which agrees well with the reported phase transition sequence from NaCl‐type phase III to phase IV (*Cmcm*) to phase V (*bcc*).^[^ ^28^, ^29^, ^34^ ^]^ Figure 2b summarizes the Raman peak positions as a function of pressure crossing from phase I to phase V. The crystal structures and corresponding pressure ranges are listed in **Table** **1**, along with previous reports from theoretical calculations and experimental results.^[^ ^27^, ^28^, ^29^, ^32^, ^33^, ^34^ ^]^ The *ω*_1_ and *ω*_2_ Raman peaks in the zinc‐blende phase showed red‐shift with increasing pressure, indicating a continuous phonon softening of the TO mode until the phase transition from zinc‐blende to cinnabar occurred at 1.6 GPa accompanied by a volume collapse.^[^ ^32^ ^]^ The Raman peaks *ω*_1_ − *ω*_2_ displayed monotonically blue‐shift up to 41 GPa crossing from phase II to V, indicating an increasing strength of these Raman vibrations with pressure. More interestingly, the phonon mode of *ω*_3_ displayed blue‐shift first and then red‐shift with pressure above 3.1 GPa within phase II.

**Table 1:** High pressure structures and their pressure ranges of HgTe

Structure	Pressure range [GPa]		
	Theory (ref. ^[^ ^34^ ^]^)	Experiment(refs. ^[^ ^27^, ^28^, ^29^, ^32^, ^33^ ^]^)	This work
ZB (I)	<1.5	<1.4	<1.6
Cinn (II)	1.5–8	1.4–8	1.6–8.6
NaCl‐type (III)	8–12	8–12	8.6–12.7
*Cmcm* (IV)	13.7–44.7	12–28	12.7–27.1
*Bcc* (V)	>44.7	>28	>27.1

### 2.3 Electrical Transport Characterization

A HgTe thin film with the substrate was loaded into a DAC with a four‐probe conduction arrangement. The resistance as a function of pressure at room temperature is shown in Figure S1 in the Supporting Information up to 54 GPa, which is consistent with previous literature reports.^[^ ^35^, ^36^ ^]^ The resistance changes discontinuously at 1.6, 7.8, 11.6, and 27.3 GPa, corresponding to the onset transition pressures from phase I to V. A prominent feature of five orders of magnitude higher resistance in the pressure range of 1.6–3.9 GPa (first half of cinnabar phase) followed by a sharp drop indicates an electronic structure transition, consistent with the *ω*_3_ anomaly in the Raman measurement.

**Figure 3:**
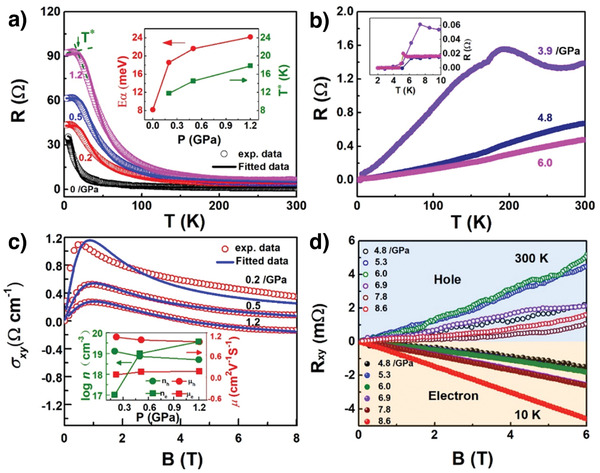
Electric transport measurements of HgTe under high pressure and low temperature. a) Resistance as a function of temperature at 0 to 1.2 GPa. Inset shows the activation energy (*E*_*α*_) and transition temperature T* as a function of pressure. b) The evolution of resistance as a function of temperature at various pressures. The inset shows the superconducting transition at 3.9, 4.8, and 6 GPa. c) Magnetic field dependence of Hall conductivity up to 8 T at various pressures, and the fitting curves (the solid blue lines) using a two‐band model. Inset: Carrier density and mobilities as a function of pressure below 1.2 GPa. d) Hall effect measurements from 4.8 to 8.6 GPa at two temperatures, 10 and 300 K. The circular and triangle symbols display the measurements at 300 and 10 K, respectively.

In **Figure** **3** and Figure S2 in the Supporting Information, we display all *R*(*T*) up to 54 GPa and temperature from 2 to 300 K measured with a physical property measurement system (PPMS, DynaCool, Quantum Design Corp.). Figure 3a shows the *R*(*T*) plots within the zinc‐blende phase with a saturated feature at ≈20 K and below. More specifically, the entire *R*(*T*) data can be fitted with a bulk and surface model. At low temperature, the resistance is dominated by the topological surface component. The metallic surface conductance *G*_sur_=1/(*A* + *BT*)  is used to fit the curves, where *A* accounts for the static disorder scattering and *B* reflects the phonon scattering.^[^ ^37^, ^38^, ^39^ ^]^ As the temperature rises, the thermally activated bulk conductance can be fitted with the Arrhenius equation as *G*_bul_(*T*) =1/*R*_b0_ e^Δ/*kT*^, where *R*_b(0)_ is the fitting parameters and Δ is the activation energy. The activation energy Δ increases from 8 to 24 meV when pressure changes from 0.2 to 1.2 GPa, as displayed in the inset of Figure 3a. In addition, the transition temperature T* from the surface state to the bulk state in the transport measurement increases as the pressure increases.

When pressure exceeds 1.6 GPa, HgTe enters into the cinnabar phase and the resistance increases dramatically, going beyond the PPMS measurement capability. When the pressure reaches 3.9 GPa, the *R*(*T*) shows a semiconductor‐to‐metal transition at ≈180 K, which is similar to the anomaly *R*(*T*) behavior observed in HfTe_5_ and ZrTe_5_.^[^ ^25^, ^26^ ^]^ The pressure‐induced superconducting transition emerged at 6.5 K (Figure 3b). The onset superconducting transition temperature (*T*_c_^onset^) decreases to around 6 K at 4.8 GPa, as shown in the inset of Figure 3b. *T*_c_^onset^ is defined as the intersection of the extension of the normal state resistance and the falling slope of *R*(*T*), as shown in Figure 3b.

Figure S2a,b depicts the Hall resistivity *ρ*_*xy*_ as a function of magnetic field *B* for different pressure at 2 and 300 K in the ambient phase, respectively. The value of *ρ*_*xy*_(*B*) changes from negative to positive (Figure S2a, Supporting Information) crossing 4.4 T at pressure of 1.2 GPa and temperature of 2 K. In addition, at 300 K, the sign for *ρ*_*xy*_(*B*) changes at 3.7 and 4.2 T under 0.3 and 1.2 GPa, respectively. These indicate a two‐band effect takes place, where both electrons and holes contribute to the electrical transport properties, similar to MoAs_2_.^[^ ^40^ ^]^ More specifically, the nonlinear behavior of Hall resistance can be well described by a two‐band model with one electron‐ and one hole‐band.^[^ ^41^ ^]^ As shown in Figure 3c, the magnetic field dependence Hall conductivity at 2 K is very consistent with the two‐band given by following equation

(1) $$\sigma_{xy}=\;eB\left[\frac{n_{h}\mu_{h}^{2}}{1+{\left(\mu_{h}B\right)}^{2}}-\frac{n_{e}\mu_{e}^{2}}{1+{\left(\mu_{e}B\right)}^{2}}\right]$$

here *n*_e_ (*n*_h_) and μ_e_ (μ_h_) represent the electron (hole) carrier density and mobility, respectively.

The pressure dependence of the carrier density and mobility of both the electron‐ and hole‐carriers is presented in the inset. We noticed that the mobility of the electron‐ and hole‐carrier is comparable, while their carrier densities show opposite trends. The Hall resistance (*R_xy_*) at 10 and 300 K in the cinnabar pressure range is plotted in Figure 3d. In the cinnabar phase, the linear field dependence of *R_xy_* dominates, suggesting that the two‐band effect does not play a role at this phase. At 300 K, HgTe maintains a p‐type dominant carrier in the pressure range of 5.3–8.6 GPa, consistent with the report from Hu et al.,^[^ ^35^ ^]^ while at 10 K, HgTe shows a negative Hall coefficient, indicating that the electron contribution becomes predominant. The transition temperature of carrier type is determined by the temperature dependence of the Hall resistance at different magnetic fields and different pressures, as shown in Figure S3 in the Supporting Information. The p‐n switching temperature increases with increasing pressure and reaches its highest value of 47 K at 7.9 GPa, then decreases slightly to 36 K at 8.6 GPa. At higher pressures, only the electron carrier exists and no more carrier‐type switching was observed. In addition, we calculated the carrier concentration of the superconducting phase at 10 K, as shown in Figure S4 in the Supporting Information. The carrier concentration is in the order of 10^22^ to 10^24^ cm^–3^, which is similar to that of metal samples.

**Figure 4:**
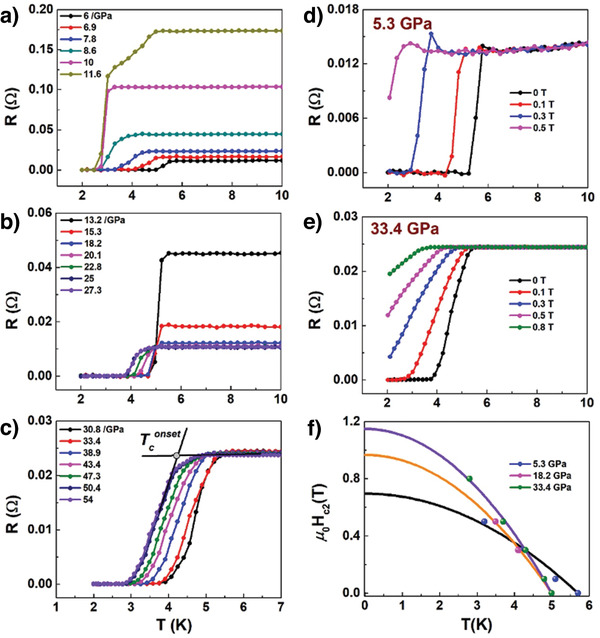
a) Resistance of HgTe single‐crystal film at various applied pressures (5.3 to 11.6 GPa) as a function of temperature. b) 13.2 to 27.1 GPa. c) 30.8 to 54 GPa. The superconducting transitions of HgTe film with an applied magnetic field *H* at d) 5.3 GPa in phase II and e) 33.4 GPa in phase V. f) The temperature dependence of upper critical field at different pressures. The solid lines represent the *G*–*L* fitting curve and spheres are experimental data.

The *R*(*T*) measurements up to 54 GPa show the superconducting behavior remains in all these high‐pressure phases (phase III–V). Zhang et al.^[^ ^42^ ^]^ calculated the occupied bands and topologic invariants in all HgTe high‐pressure phases as listed in Table S1 in the Supporting Information, where the cinnabar phase is an insulator, but the other four phases have a topological surface state.^[^ ^42^ ^]^ **Figure** **4** shows the *R*–*T* curves at higher pressures up to 54 GPa (see large temperature range in Figure S5, Supporting Information). The onset superconducting temperature *T*_c_^onset^ starts ≈6.5 K at 3.9 GPa, and decreases monotonically until it reaches a minimum of 4.5 K at 7.8 GPa with an almost linear slope of −0.5 K GPa^−1^. Then, HgTe enters into a narrow metallic phase. The *T*_c_^onset^ shows a noticeable reduction to 3.6 K at 8.6 GPa, where the phase transition from phase II (cinnabar) to III (NaCl phase) takes place. When the pressure exceeds 11.6 GPa, the HgTe enters phase IV with a jump of the onset superconducting transition temperature to 5 K.

The *R*(*T*) curve also shows the remaining transition feature of phase III with a secondary transition at 3 K (Figure 4a). A similar two‐step‐like transition can also be recognized at 13.2 GPa, which is similar to the phenomenon in ZrTe_5_,^[^ ^25^ ^]^ implying two superconducting phases coexist in this pressure. Based on our Raman data and high‐pressure XRD by Huang and Ruoff,^[^ ^43^ ^]^ both superconducting phases might coexist between 11.6 and 13.2 GPa in HgTe.

When pressure is above 13.2 GPa, phase III fully transfers into phase IV and the *T*_c_^onset^ henceforth decreases monotonically with pressure, as shown in Figure 4b. Above 30.8 GPa, HgTe enters into phase V with a jump of the starting *T*_c_^onset^ to 5.1 K followed by slowly decreasing to 54 GPa, the highest pressure studied in this work (Figure 4c). For demonstrating the superconducting transition clearly, we measured the *R*–*T* curves under various magnetic fields at representative pressures of 5.3 and 33.4 GPa. The evolutions of the *T*_c_^onset^ as a function of the applied magnetic field are plotted as the insets in Figure 4d,e. The corresponding upper critical fields are plotted as a function of temperature and shown in Figure 4f. The *T*_c_^onset^ was gradually suppressed by the increasing magnetic field, and the *H*_c_(*T*) can be calculated using Ginzburg–Landau theory, $H_{C}\;\left(T\right)=H_{C}\;\left(0\right)\left[1-{\left(\frac{T}{T_{C}}\right)}^{2}\right]$. The upper critical field *H*_c2_(0) is estimated to be 0.7 T at 5.3 GPa in phase II, 1.0 T at 18.2 GPa in phase IV, and 1.1 T at 33.4 GPa in phase V. It is worth noting that this is well below the Pauli–Croston limit, which is determined by µ_0_ *H*_p_ [T] = 1.84*T*_c_ [K].

### 2.4 Density Functional Theory (DFT) Calculation on the Band Structure

**Figure 5:**
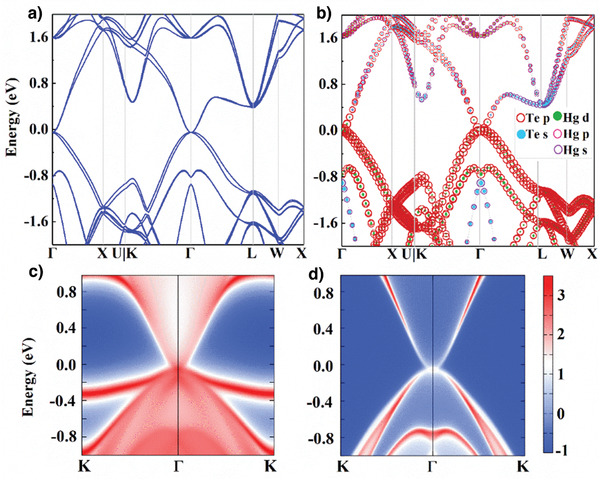
The DFT calculations for the electronic band structures and surface states of HgTe film in the zinc‐blende phase. a) 2D band structure and b) fat band for HgTe under 1 GPa pressure, where the size of the dots is proportional to the weight contributed by the different orbitals of each element. c) Surface state spectrum (SSS) and d) only bulk HgTe is present in the SOC for the 010 surface.

As shown above, our electrical transport measurements reveal the independence of the metallic surface and bulk transport characters at low temperature in phase I, a typical TI character. To better understand the topological property of HgTe, we performed the DFT calculations on the electronic band structures and surface states, as shown in **Figure** **5**. The band structures of zinc‐blende HgTe under pressure of 1 GPa with spin–orbit coupling (SOC) is presented in Figure 5a, where a dispersion curve similar to a bulk 3D Dirac cone is present at the high symmetry Γ point in the Brillouin zone. The fat band structure of HgTe is presented in Figure 5b, the size of the circle is proportional to the weight of the related orbital contribution in HgTe. The p states of the Te atoms are made almost entirely of the Dirac point and nearby band, meanwhile, the contribution of the Hg d orbital is very small. To identify the topological properties of zinc‐blende HgTe, we obtained the *Z*_2_ topological invariant by calculation. The *Z*_2_ number for 3D bulk materials can be expressed as (*ν*_0_, *ν*_1_, *ν*_2_, *ν*_3_) from the *Z*_2_ calculations of six‐time reversal invariant planes.^[^ ^4^ ^]^ *ν*_0_ = 1 shows that bulk is a strong topological material in all three‐reciprocal lattice directions, while *ν*_0_ = 0 indicates a weak topological material.^[^ ^4^ ^]^ For the zinc‐blende phase, the calculation gives the *Z*_2_ number of (1 000) implying a strong topological material. Additionally, the calculated surface state spectra (SSS) for the 010 surface are shown in Figure 5c. The calculated SSS exhibits linear band dispersion in the vicinity of the Fermi level. Figure 5d shows only the bulk's contribution to the band structure. Figure 5c‐d shows that the contribution of the bulk is minimal around the Fermi level, but the contribution of the surface state near the Fermi surface is very significant. Here, measurements of the electrical transport characterization and comparison with band‐structure calculations indicate that zinc‐blende HgTe is a TIs.

## 3 Discussions

**Figure 6:**
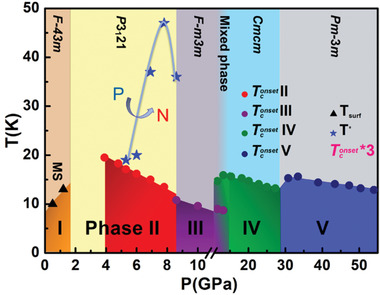
Temperature–pressure structure phase diagram of HgTe. The triangle symbols denote the *T*_surf_, the temperature of the saturated resistance displays the metal surface state (MS). The circles represent the *T*_c_^onset^ values extracted from electrical resistance measurements. The blue stars (T*) are the carrier switching temperatures derived from the Hall measurements. For clarity, the value of the *T*_c_^onset^ here is multiplied by a factor of 3.

The structure and superconducting phase diagrams in the *P*–*T* space are summarized and plotted in **Figure** **6**. In phase I (*F*‐43*m*, zinc‐blende), the characteristic surface‐dominated temperature *T*_surf_ from topological structure increases with applied pressure and vanishes once the first high‐pressure phase occurred, with an insulator cinnabar phase (*P*3_1_21, phase II) emerging. This increase in *T*_surf_ is explained by the pressure enhancement of the bandgap with the zinc‐blende structure. As in the Raman spectra under high pressure, the blue‐shift of the *ω*_3_ mode between 1.6 and 3.1 GPa shows a general stiffening of the regular compression effect as predicted by the phonon‐dispersion calculation,^[^ ^44^ ^]^ while the red‐shift of *ω*_3_ mode (softening) afterward is concurrent with the emergence of superconductivity. This is similar to cuprates’ superconducting behavior when the phonon softening occurs.^[^ ^45^, ^46^ ^]^ The *ω*_3_ mode origins from a two‐phonon combination of the type TO and transverse acoustic (TA).^[^ ^47^ ^]^ The abnormal frequency shift of *ω*_3_ mode is attributed to the bandgap decreasing with the applied pressure.^[^ ^48^ ^]^ In general, the decrease of the bandgap with pressure in semiconductors is from the change of bandwidth by increased overlap interaction with decrease of the atomic distance. At the same time, the softening of *ω*_3_ mode actually could be related to the change in the electronic structure transition.

From the view point of crystallography, HgTe is with fourfold coordination in zinc‐blende structure (Phase I) and gradually changed to sixfold coordination in rocksalt structure (Phase III) by passing through the cinnabar structure (Phase II). The gradual change of fourfold coordination to sixfold coordination may lead to the decrease of valence electrons and the obvious increase of holes, resulting in a sign change of the carrier charges as an increase of pressure. Just after the entrance of cinnabar phase, an obvious transition from electron‐ to hole‐dominated transport is observed as a function of temperature. The carriers' charge sign from positive to negative with decrease of temperature suggests a complex Fermi surface. Closely looking at the electronic band structure of HgTe under high pressure in cinnabar structure, we found the valence bands are mainly contributed by Te p states, while the conduction bands are formed from both Hg s and Te p states.^[^ ^49^ ^]^ The carriers' charge sign change suggests a complex Fermi surface, which may be explained by possible topological phase transition (with an energy gap opening at the intersection), multi‐band pressure‐induced competition, or pressure‐induced Lifshitz phase transition.^[^ ^25^, ^50^ ^]^ The emerging of superconducting is observed at temperature of ≈6.5 K and pressure of 3.9 GPa, where the carrier is transformed into n‐type and persisted in all high‐pressure phases thereafter. There still is possibility that unconventional superconductivity can exist in the Pauli–Croston limit.^[^ ^51^ ^]^ As shown in the case of pyrochlore superconductor KOs_2_O_6_, *H*_C2_(0) is much lower than *H*_P_, which is due to the missing spatial inversion symmetry.^[^ ^51^ ^]^ Therefore, it is possible that the superconductivity in HgTe under high pressure be entangled in topological states. However, the relationship between superconductivity and topological property in high pressure phase of HgTe needs to be further explored, such as by using different strategies under high pressure, like gating, intercalation, reducing dimensions, doping, and the near neighbor effect.

## 4 Conclusion

By subjection to the hydrostatic pressure, we explore the pressure tuning effects on crystal structure and electronic structure of HgTe in a 400 nm thick single‐crystal sample by means of resistivity, Hall effect, and Raman spectroscopy. The emergency of pressure‐induced superconductivity was observed with the highest *T*_c_^onset^ ∼ 6.5 K at 3.9 GPa after an electronic transition to metallic state in the cinnabar phase, and confirmed with Band structure calculations. From 3.9 to 54 GPa, the HgTe undergoes four high pressure phases with superconducting property and increasing upper critical field from 0.7 to 1.1 T. In phase I and II, the competition of hole and electron carriers leads to carrier type switch in the temperature ranged from 19 to 47 K. Phase III–V all behave as metal and only electron carrier dominates the transport property. Combining DFT calculation and comprehensive structure and transport measurements, high pressure HgTe study demonstrates a great potential material for exploring the relationship of topological structure and superconductivity.

## 5 Experimental Section

### Single‐Crystal Film Growth and Characterization

Single‐crystal film of HgTe (111) of 400 nm thick was grown on a GaAs(100) substrate with CdTe(111) buffer layer by MBE (DCA450).

### High‐Pressure Raman spectroscopy

High‐pressure Raman spectra were measured using a symmetric DAC with culet size of 300 µm. A T301 stainless‐steel gasket was used and preindented to 40 µm in thickness. A 100 µm hole was drilled in the center of the gasket severing as sample chamber. HgTe film with substrate was polished to a total thickness below 30 µm (HgTe layer + CdTe layer + GaAs substrate) and loaded inside the gasket hole with a ruby microsphere (for pressure measurements). Silicone oil was used as the pressure‐transmitting medium. The Raman experiments were performed in a Renishaw inVia Raman microscope equipped with a 2400 line mm^−1^ grating. The excitation laser had wavelength of 532 nm and was focused down to a 10 µm spot (full‐width at half‐maximum). The pressure was determined by the ruby fluorescence method.^[^ ^52^ ^]^

### High‐Pressure Transport Measurements

The transport properties of HgTe single‐crystal film were measured using the standard four‐probe method in DAC made by nonmagnetic BeCu alloy. Pressure was generated by a pair of diamond anvils with culet size of 300 µm. A nonmagnetic stainless‐steel gasket was preindented to 40 µm and drilled a hole in the center with 180 µm in diameter. The gasket was then covered by cubic BN insulation layer to protect electrode from short circuit with the conducting gasket. A smaller center hole was then drilled in the insulating layer to serve as sample chamber. The HgTe film with a dimension of 100 µm × 100 µm × 30 µm was loaded into sample chamber with silicone oil as pressure transmitting medium. Gold foils of 5 µm in thickness were used as electrodes. For fundamental details of electric transport measurements, see Figure S6, Supporting Information. The PPMS (Dynacool, Quantum Design Inc.) was used to perform the electric transport experiments.

### Band Structure Calculations

All first‐principles calculations reported in this work were performed by DFT^[^ ^53^ ^]^ as implemented in the Vienna ab initio simulation package (VASP),^[^ ^54^ ^]^ using projector augmented wave pseudo potentials and the Ceperley–Alder local‐density approximation exchange and correlation functional.^[^ ^55^ ^]^ The kinetic energy cutoff for the plane wave basis was set to be 500 eV. The Brillouin zone was sampled with Monkhorst–Pack *k*‐point grid of 11 × 11 × 11. For instance, all the structures were fully relaxed with a force on each atom that was less than 0.02 eV Å^−1^. The convergence threshold for energy was fixed at 10^–6^ eV. The SOC effect was taken into account in this work as it was essential for heavy elements. The topological property was calculated by using WannierTools^[^ ^56^ ^]^ depended on tight‐binding (TB) model, construct TB model by Quantum Espresso (QE),^[^ ^57^ ^]^ and wannier90^[^ ^58^ ^]^ as a post‐processing tool to generate the TB file.

## Conflict of Interest

The authors declare no conflict of interest.

## Author Contributions

Q.F.Z., J.Z., and Q.L. contributed equally to this work. X.M.Q., W.G.Y., J.L.Z., P.P.C., Q.F.Z. proposed and designed the research; J.Z. and P.P.C. contributed in sample growth; X.Y.Z., W.Y.G., J.M.C., and X.Y. contributed to the band structure calculations; Q.F.Z. and Q.L. performed Raman experiments; Q.F.Z., Q.L., M.L.J., and N.N.L. performed the electrical transport measurements; W.Z.S., F.F.W., T.W., M.L.J., N.N.L., Y.H.W. contributed to maintenance of experimental equipment and providing technical supporting. Q.F.Z. and X.M.Q. wrote the manuscript with input from all the co‐authors.

## Supporting information

Supporting Information

## Data Availability

The data that support the findings of this study are available in the supplementary material of this article.
