# Supplementary material for: Pressure‐Induced Superconductivity in HgTe Single‐Crystal Film
Source: Adv Sci (Weinh). 2022 Apr 25;9(18):2200590. doi: 10.1002/advs.202200590 (PMC9218769; doi:10.1002/advs.202200590)
Supplement: Supplementary file 1 — Supporting Information [file ADVS-9-2200590-s001.pdf]

ADVANCED SCIENCE
Open Access

Supporting Information

for *Adv. Sci.*, DOI 10.1002/advs.202200590

Pressure-Induced Superconductivity in HgTe Single-Crystal Film

Qiang Li, Jian Zhang, Qunfei Zheng, Wenyu Guo, Jiangming Cao, Meiling Jin, Xingyu Zhang, Nana Li, Yanhui Wu, Xiang Ye, Pingping Chen, Jinlong Zhu*, Tao Wang, Wangzhou Shi, Feifei Wang, Wenge Yang* and Xiaomei Qin**

Supporting Information

Title

Pressure-induced superconductivity in HgTe single crystal film

Pressure-induced superconductivity in HgTe single crystal film

Qiang Li, Jian Zhang, Qunfei Zheng, Wenyu Guo, Jiangming Cao, Meiling Jin, Xinyu Jiang, Nana Li, Yanhui Wu, Xiang Ye, Pingping Chen, Jinlong Zhu*, Tao Wang, Wangzhou Shi, Feifei Wang, Wenge Yang*, Xiaomei Qin**

Q. Li, Q. Zheng, W. Guo, J. Cao, Y. Wu, Prof. X. Ye, Prof. T. Wang, Prof. W. Shi, Prof. F. Wang, Prof. X. Qin
Department of Physics, Shanghai Normal University, Shanghai 200234, China
E-mail: xmqin@shnu.edu.cn
Q. Li, Q. Zheng, Dr. M. Jin, Dr. N. Li, Prof. J. Zhu, Prof. W. Yang
Center for High Pressure Science and Technology Advanced Research (HPSTAR), Shanghai 201203, China
E-mail: zhujl@sustech.edu.cn, yangwg@hpstar.ac.cn
Dr. J. Zhang, Prof. P. Chen
State Key Lab of Infrared Physics, Shanghai Institute of Technical Physics, Chinese Academy of Sciences, Shanghai 200083, China
E-mail: ppchen@mail.sitp.ac.cn
Prof. X. JiangProf. J. Zhu
Department of Physics, Southern University of Science and Technology, Shenzhen, 518055 China

[#] Contributed equally to this work

*Corresponding authors: ppchen@mail.sitp.ac.cn; zhujl@sustech.edu.cn;

yangwg@hpstar.ac.cn; xmqin@shnu.edu.cn.

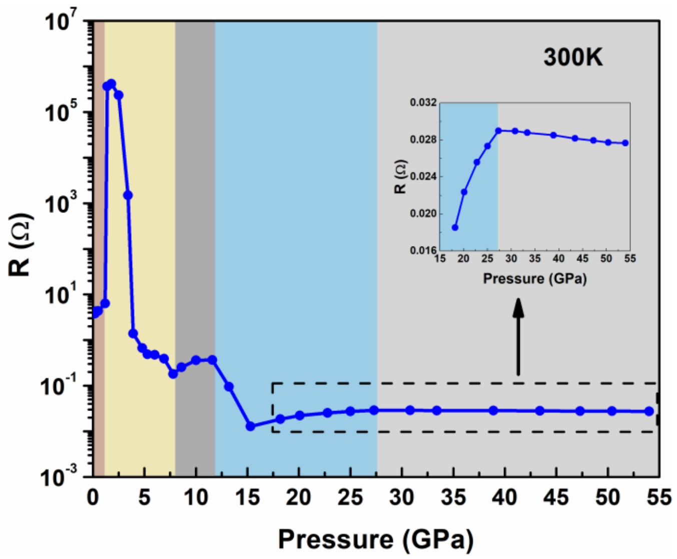

Figure S1. Resistance of HgTe as a function of pressure at 300 K. The inset displays the resistance above 18 GPa.

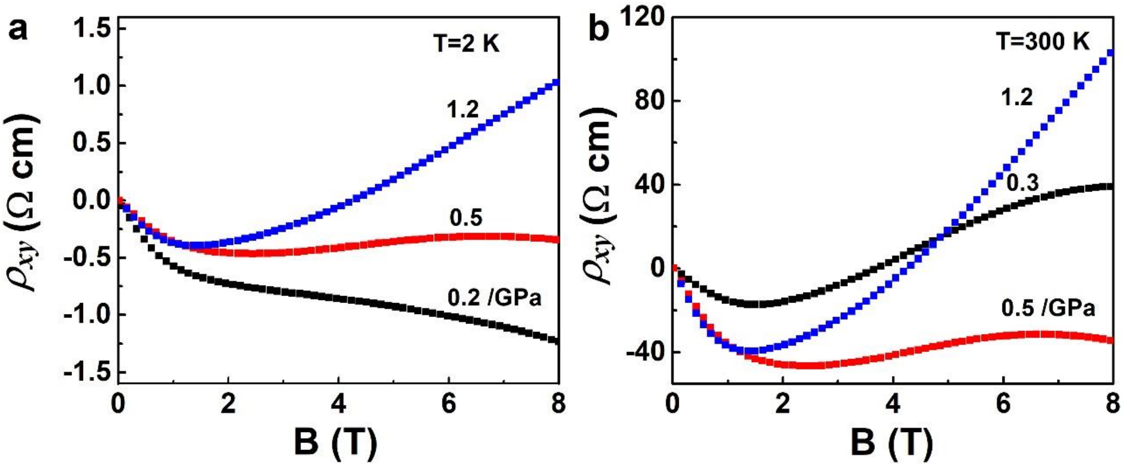

Figure S2. The Hall resistivity ρ_{xy} as a function of magnetic field for different pressure at 2 K (a) and 300 K (b).

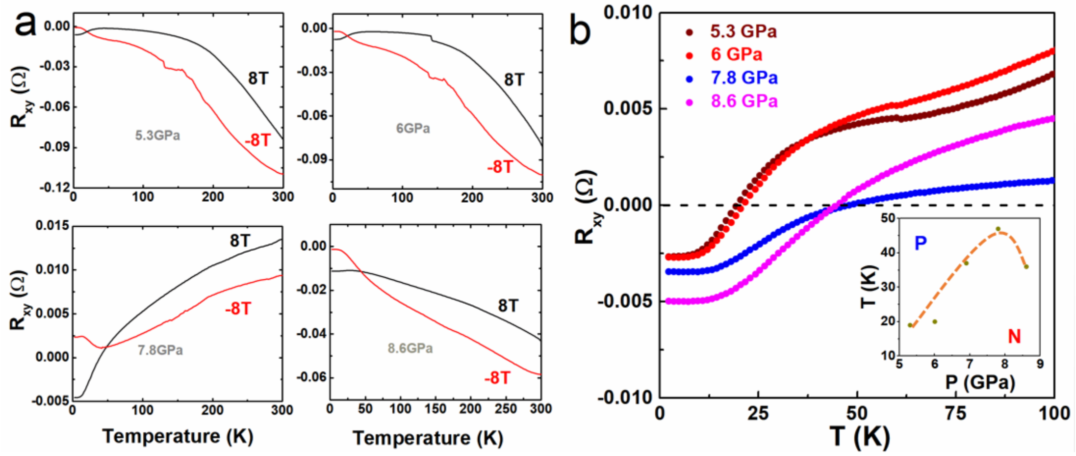

Figure S3. (a) The temperature dependence of the Hall resistance at 8 T and -8 T at pressure between 5.3 GPa and 8.6 GPa. (b) The temperature dependence of the Hall resistance. The inset shows the P-N transition of HgTe film in the temperature and pressure phase diagram.

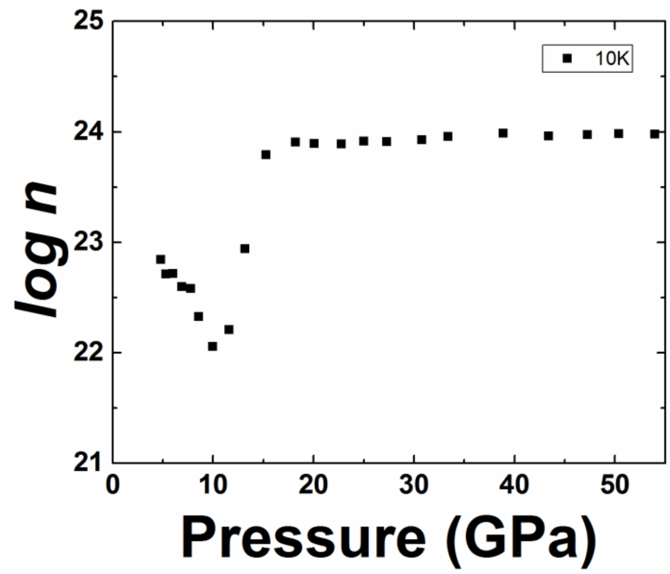

Figure S4. The pressure dependence of the carrier concentration at 10 K.

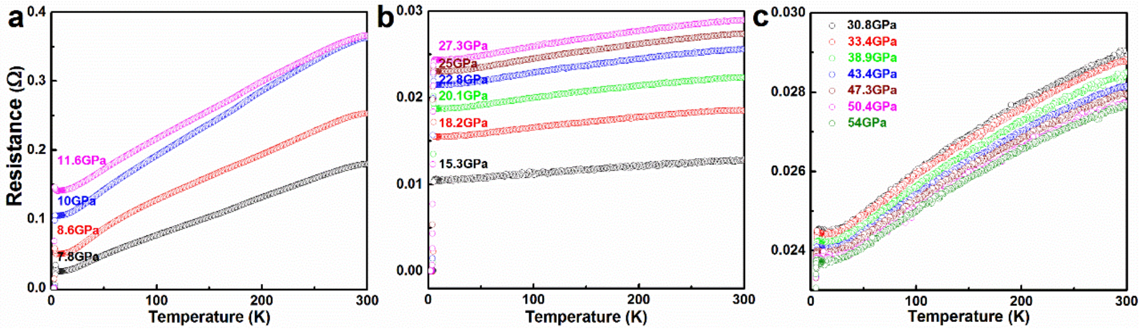

Figure S5. The evolution of HgTe resistance as a function of temperature and pressure.

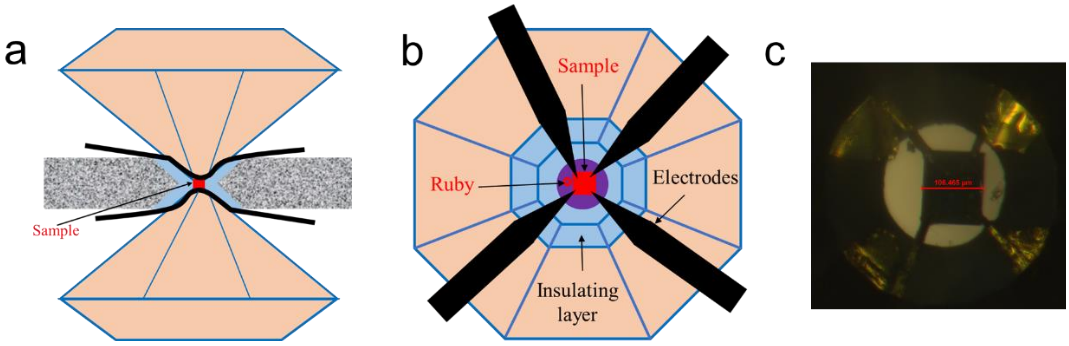

Figure S6. (a) and (b) display side- and top-view schematic diagrams of the diamond anvil cell (DAC), showing the diamond anvils, the sample chamber (purple), the insulating cubic BN layer (Bule), the sample (red), a ruby microsphere (red ring), a nonmagnetic stainless-steel gasket (silver) and four electrodes for the transport measurements. (c) Optical micrograph of a rectangular sample and four gold electrodes in a DAC.

Table S1. Topological Class of HgTe at high pressure, according to the algorithm developed by Zhang *et al* (*57*).

HgTe	Space Group Number	Space Group Symbol	Topological Class
ZB (I)	216	*F*-43*m*	High symmetry point semi-metal
Cinnabar (II)	152	$P3_121$	Trivial insulator
NaCl-type (III)	225	*Fm*-3*m*	High symmetry point semi-metal
Cmcm (IV)	63	*Cmcm*	High symmetry line semi-metal
BCC (V)	221	*Pm*-3*m*	High symmetry line semi-metal
